# Behavioral weather insurance: Applying cumulative prospect theory to agricultural insurance design under narrow framing

**DOI:** 10.1371/journal.pone.0232267

**Published:** 2020-05-01

**Authors:** Tobias Dalhaus, Barry J. Barnett, Robert Finger

**Affiliations:** 1 Agricultural Economics and Policy Group, ETH Zürich, Zürich, Switzerland; 2 Department of Agricultural Economics, University of Kentucky, Lexington, Kentucky, United States of America; Universidad de Granada, UNITED KINGDOM

## Abstract

Experience across many countries shows that, without large premium subsidies, crop insurance uptake rates are generally low. In this article, we propose to use the cumulative prospect theory to design weather insurance products for situations in which farmers frame insurance narrowly as a stand-alone investment. To this end, we introduce what we call “behavioral weather insurance” whereby insurance contract parameters are adjusted to correspond more closely with farmers’ preferences. Depending on farmers’ preferences, we find that a stochastic multiyear premium increases the prospect value of weather insurance, while a zero deductible design does not. We suggest that insurance contracts should be tailored precisely to serve farmers’ needs. This offers potential benefits for both the insurer and the insured.

## 1. Introduction

Climate risks threaten agricultural crop production and are expected to become even more pronounced due to climate change [1]. Crop insurance could be one of the key risk management tools to help address increased weather variability resulting from climate change [2, 3]. In many countries, crop insurances are heavily subsidized to encourage farmers to participate. In fact, premium subsidies are often so high that taking out insurance has a positive expected value [4,5,6,7]. In contrast, the uptake of unsubsidized crop insurance is often low (See note 1 in S1 Footnotes [8]). Assuming a standard expected utility (EU) framework, this observation is not consistent with the optimal behavior of risk averse farmers. A possible explanation for this anomaly is that some farmers do not assign insurance premiums and payouts to fluctuations in crop income, but tend to frame insurance narrowly as a stand-alone investment [9,10,11]. Recent evidence also suggests that the cumulative prospect theory (CPT) [12] may be a better predictor of farmers’ insurance decision-making than EU theory [6,7,9,10,11,13]. For these farmers, losses are felt whenever premiums exceed payouts while gains are perceived in the opposite case.

In this paper, we investigate the feasibility of adjusting insurance contracts so that they fit better within a stand-alone investment framing. Therefore, we assume that some farmers frame insurance purchase decisions as a stand-alone investment rather than a risk management tool (See note 2 in S1 Footnotes). We propose to modify the traditional weather insurance (TWI) design and introduce what we call behavioral weather insurance (BWI). To this end, we propose a two-step design of weather insurance products. Firstly, the BWI should be effective in reducing farmers’ financial exposure to weather risk. Secondly, the design of this BWI should be adjusted in line with insights derived from CPT, thus extending the narrower EU framework.

Our study enlarges the available literature in three dimensions. Firstly, it reviews currently available literature on crop insurance decision-making under narrow framing and CPT preferences [9,10,11] and explicitly simulates and tests how contract adjustments can make insurance more attractive under such framing (see e.g. Sydnor [14] on behavioral decision-making in home insurance). Secondly, it adds to the literature on optimal crop insurance which at present focuses mainly on expected utility maximizing decision-making [15,16]. Thirdly, our BWI is a first attempt to optimally design crop insurance under behavioral decision-making, something which others are attmepting in sectors outside of agriculture [e.g. 17,18].

Based on a microeconomic framework for insurance decision-making under narrow-framing with cumulative prospect theory preferences, we derive two adjustments to traditional weather insurance contracts and introduce the behavioral weather insurance that has the potential to increase the prospect value. Firstly, Adjustment 1 proposes “Insure small losses also (no deductible)”. Secondly, Adjustment 2 proposes “Conclude a multiyear contract and only pay premiums in years of no crop losses or, if there are no years with no losses, at the end of the contract period”.

We use 15 years of yield data from 38 representative winter wheat producers in the eastern part of Germany. This is one of Europe’s most productive grain growing regions, but it is also extremely drought-prone. We match yield data with high-resolution rainfall and phenology data and tailor drought (i.e. lack of rainfall) index insurance contracts (TWI and BWI) for each farm. We are thus able to simulate the insured and uninsured revenue stream over the observed time period based on hypothetical insurance payouts and premium payments. We apply a two-step test procedure. Firstly, we test whether both TWI and BWI can effectively reduce the insured party’s financial exposure to production risk. To this end, we test for increases in expected utility (EU) across various scenarios of risk aversion and along quantiles of the revenue distribution between insured and uninsured revenues. Secondly, we evaluate potential changes in insurance demand through BWI compared to TWI by testing for prospect value changes under various real world elicited CPT specifications and the assumption of a stand-alone investment framing (see Bocquého, Jacquet, and Reynaud, [19] and Bougherara et al. [20] for elicited CPT preferences).

Our results show that BWI with Adjustment 1 only “Insure small losses also (no deductible)”, increases the risk reducing capacity in Step 1, but does not increase the prospect value under narrow framing in Step 2. In contrast, our findings reveal that compared to TWI, BWI with Adjustment 2 only (“Conclude a multiyear contract and only pay premiums in years of no crop losses or, if there are no years with no losses, at the end of the contract period”) can increase an insurance’s prospect value under narrow framing, while preserving the risk reducing capacity. This added value opens new possibilities for making crop insurance more attractive, apart from subsidization. The unexploited potential of behavioral insurance, which has proved useful in other branches, offers valuable opportunities for both insurers and insured parties to strengthen the resilience of the agricultural sector against extreme weather events. Our findings lay a foundation also for other framings, such as state-dependent reference levels [13, 7] that can be integrated into the design of crop insurances.

We proceed as follows. Firstly, the theoretical framework of EU and CPT is used to propose adjustments to the design of TWI contracts to create BWI. Secondly, we introduce hypotheses about preferences for BWI designs to be tested in relation to TWI. We assume an EU value function and then a CPT value function, according to the above two-step procedure. Thirdly, we test these hypotheses using data from a drought-prone wheat production region in eastern Germany.

## 2. Methodology

This section gives an overview of the decision-making criteria under risk, the specifications used, the underlying testing procedure and the insurance design.

### 2.1. Decision-making under risk

In the next section, we present the methodology on which our two-step approach for designing BWI is based. We begin with an overview of the EU framework used in Step 1. Step 2 describes how cumulative prospect theory is used to assess insurance from a narrowly framed stand-alone investment perspective. We then combine both frameworks within one decision-making model. On this basis, we are able to propose Adjustments to the insurance contract that potentially increase both expected utility and prospect value, thus making it more attractive for the two interest groups (expected utility maximizers and prospect value maximizers) within the farming population.

#### 2.1.1. Expected utility theory (Step 1)

In the EU framework, terminal wealth ***W***_***ti***_ for farm *i* in year *t* is transformed into a utility value using a utility function ***U***(***W***_***ti***_). The occurrence probability weighted average of these is the expected utility ***E***[***U***(***W***_***ti***_)] (EU). For the sake of clarity in our analysis, we assume that farmers produce wheat only, resulting in terminal wealth ***W***_***ti***_ to follow ***W***_***ti***_ = ***δy***_***ti***_ + ***π***_***ti***_ − **Γ**_***i***_. Here, ***y***_***ti***_ denotes the yield of farm *i* in year *t*, ***π***_***ti***_ the insurance payout and **Γ**_***i***_ the insurance premium (see note 3 in S1 Footnotes). The standard assumption is that farmers chose their insurance plans according to the expected utility maximization problem, i.e. **max *E***[***U***(***W***_***ti***_)]. If farmers are downside risk averse, insurance payouts ***π***_***ti***_ should cover downward movements of stochastic yields ***y***_***ti***_. Thus, assuming that insurance premium **Γ**_***i***_ is fair, and all else being equal, changes in ***E***[***U***(***W***_***ti***_)] through modified insurance plans serve as proxy for changes in welfare and consequently for changes in the ability of the respective insurance to reduce the financial exposure to risks.

For this analysis, we use a power utility function to reflect farmers’ preferences [21].

$${\text{U}}_{ti\varphi }(W_{ti})=\{\begin{array}{c} \frac{W_{ti}^{1-\varphi }}{1-\varphi }\;if\;\varphi \neq 1\\ \text{ln}\left(W_{ti}\right)\;if\;\varphi =1 \end{array}$$ (1)

where *φ* is the measure of relative risk aversion. As a result, we obtain vectors *eu*_*κφi*_ containing EU values for each of the *i* farms for the two insurance designs *κ* (*κ = TWI* or *BWI*) and levels of risk aversion are φ.

Consequently, changes in *E*[*U*(*W*_*ti*_)] can likewise be expressed as changes in the willingness to pay to eliminate risks. Thus, any increase in *E*[*U*(*W*_*ti*_)] can be displayed as a decrease in the risk premium *R*. Furthermore, insurance premium Γ_*i*_ shall not exceed a farmer’s individual risk premium *R*, which constitutes the maximum amount a farmer is willing to pay to eliminate the risk arising from *y*_*it*_ and which is dependent on his risk preferences. Based on Kim et al. [22], we decompose *R* into incremental risk premiums Δ*R*_*k*_, i.e. “the incremental willingness to pay to eliminate the risk in the k-th quantile, moving it to the mean payout, while risk has been already eliminated in lower quantiles”. As a result, we obtain vectors Δ*R*_*kκφi*_ containing the incremental risk premium of farm *i*, quantile *k*, insurance design *κ* and levels of risk aversion φ. We are thus able to decompose changes in the financial risk exposure, with and without insurance, into parts of the wealth distribution. See the online supplementary file for further information on how we mathematically derive Δ*R*_*kκφi*_ based on quantile moments of *W*_*ti*_.

#### 2.1.2. Cumulative prospect theory (Step 2)

As farmers tend to deviate from the expected utility maximizing insurance choice, Babcock [9] suggests that they might frame insurance narrowly as a stand-alone investment and evaluate this investment, which is counter to the expected utility theory (see also [10,11]). In fact, previous studies suggest that people frequently make decisions that are contrary to EU determined preference rankings (see Stamer, [23] for an overview). Consequently, a number of alternative theories have been proposed to explain and predict human behavior, such as CPT or rank-dependent expected utility [24]. In particular, CPT has received considerable attention in recent agricultural economics literature in general (e.g. [25,26]) and crop insurance demand literature in particular [6,7,9,10,11,13].

CPT extends EU by distinguishing gains and losses as deviations from a certain reference point, resulting in two (potentially) different ‘utility’ functions combined into a value function v(*σ*), which implies risk aversion over gains and risk seeking behavior over losses:

$${\text{v}}_{ti\alpha \lambda }(\sigma_{ti})=\{\begin{array}{c} \sigma_{ti}^{\alpha }\;if\;\sigma_{ti}>0\\ 0\;if\;\sigma_{ti}=0\\ -\lambda {\left(-\sigma_{ti}\right)}^{\alpha }\;if\;\sigma_{ti}<0 \end{array}$$ (2)

Instead of terminal wealth realizations, CPT transforms single prospect outcomes *σ* into prospect values *v*, which depend on the level of risk aversion *α* and loss aversion *λ* (See note 4 in S1 Footnotes). v(*σ*) is strictly increasing and |v(*σ*)| < |v(−*σ*)| suggests loss aversion. Moreover, *∂*^2^v(*σ*)/∂*σ*^2^ ≤ 0 for *σ* > 0 (implying risk aversion in gains) and *∂*^2^v(*σ*)/∂*σ*^2^ ≥ 0 for *σ* < 0 (implying risk seeking in losses) jointly indicate diminishing sensitivity towards changes in *σ* with increasing distance from the reference point for both gains and losses [27].

We base the CPT framework on Babcock [9] and frame the EU increasing weather insurance from Step 1 as a stand-alone investment. This means that gains are perceived when payouts exceed premiums and losses are felt in the opposite case (see also Barberis, Huang & Thaler, [28] for further details on narrow framing). Babcock [9] finds that farmers tend to frame insurance so that the difference between payouts and premiums for farm *i* in year *t* is indicated by the prospect outcome *σ*_*ti*_ = *π*_*ti*_ − Γ_*i*_. The reference point *R*_*i*_ is then equal to *σ*_*ti*_ = 0. There is a corresponding probability of occurrence *p*_*ti*_ for each *σ*_*ti*._ These probabilities are translated into decision weights, allowing for the tendency observed among decision-makers to overweight small probabilities and underweight large ones [24,13], i.e. by using a function *ω*(*p*). Assuming ordered outcomes *σ*_*i*_ with probabilities *p*_*i*_ of farm *i* over the years *t* from largest loss year *m* to largest gain year *n*, the decision weight of a gain in year *t* is defined as

$$\begin{array}{c} \vartheta_{ti}^{+}=\;\omega \left(p_{t}+\cdots +p_{n}\right)\\ -\;\omega \left(p_{t+1}+\cdots +p_{n}\right) \end{array}$$

and of a loss in *t* as

$$\begin{array}{c} \vartheta_{ti}^{-}=\;\omega \left(p_{m}+\cdots +p_{i}\right)\\ -\;\omega \left(p_{m}+\cdots +p_{i-1}\right) \end{array}$$

The final prospect value *pν*_*iα*_ is evaluated by summing up the weighted single year values:

$${pv}_{i\alpha \lambda \gamma }=\sum_{t=1}^{n}{\vartheta_{ti}\text{v}}_{ti\alpha }(\sigma_{ti})$$ (3)

Thus, when insurance is framed narrowly as a stand-alone investment, the maximization problem is *max pν*_*iαλγ*_, where farmer *i* evaluates the insurance contract based on all available realizations of *σ*_*ti*_. Hence, CPT enables us to apply a second performance measure to assess insurance in addition to the risk reducing properties provided by the EU framework. This is in line with Jäntti et al. [29] who suggest the use of welfare measures according to the subject’s underlying decision-making process. More specifically, we use the prospect value to measure welfare when the subject tends towards prospect value maximization.

#### 2.1.3. Coexistence of decision-making processes and contract adjustments

In accordance with Harrison & Rutström [30], we assume that “several behavioral processes […] coexist” within the farming population (see note 5 of S1 Footnotes [31]) (see also Sproul & Michaud [32]). Hence, any number of (unobservable) decision rules could be assumed and our framework allows various decision rules to be tested. However, we will focus on the two that have been addressed most prominently in the crop insurance decision literature, namely **max *E*[*U*(*W***_***ti***_**)]** and ***max pν***_***iαλγ***_, i.e. expected utility maximization (***i***.***e***. **max *E***[***U***(***W***_***ti***_)]) and maximization of a prospect value based on cumulative prospect theory (***i***.***e***. ***max pν***_***iαλγ***_). The framework presented here allows further processes to be included if experimental evidence suggests their existence. If some of the farmers maximize ***pν***_***iαλγ***_, we advocate that insurance design should take this into account. By assuming that BWI should increase both EU and ***pν*** according to our two step-procedure, we aim to increase the welfare for both interest groups, i.e. EU and CPT maximizers [29], while keeping EU related risk reducing properties constant.

In the following, we derive Adjustments of insurance contracts that potentially increase both the expected utility *E*[*U*(*W*_*ti*_)] and the prospect value *pν*_*iαλγ*_ of weather insurance. In order to maximize *pν* of an insurance under narrow framing (See note 6 of S1 Footnotes), we recall the properties of the value function in this specific case. The diminishing sensitivity property of v(*σ*) in the gain domain, i.e. *∂*^2^v(*σ*)/∂*σ*^2^ ≤ 0 for *σ* > 0, implies that decision-makers have a particularly positive attitude towards small gains that occur close to their reference point. This property is consistent with the decreasing marginal utility property of *U*(*W*). However, in CPT this sensitivity is shifted and appears close to the reference point. In our insurance case, this implies a preference for frequent positive *σ*_*ti*_, i.e. insurance payouts frequently exceeding premiums. Moreover, the concavity of v(*σ*) in the gain domain implies risk aversion in gains, i.e. a preference for a lower variation in gains. Therefore, individuals prefer multiple small gains in relation to, or in addition to, infrequent large gains [33,34,35]. When this is applied to weather insurance, farmers exhibit a preference for insurance with a lower volatility in payouts. Hence, farmers favor contracts that provide larger payouts in the case of catastrophic losses as well as small payouts with higher frequencies. It follows that insurance without deductibles might benefit farmers’ prospect value under narrow framing. Hence, we propose the following first Adjustment to be tested.

Adjustment 1: Insure small losses also (no deductible).

Compared to a situation where only larger yield losses are insured, two differences are expected in the probability mass of payouts. Firstly, payouts occur more often. Secondly, as there are more payouts, the overall payout mean is shifted away from zero. In addition, increasing the number of payouts generates higher premiums, which, in our narrow framing example, are experienced as losses. Hence, changes in *pν* through Adjustment 1 depend on how decision-makers rate less risk in the gain domain in comparison to additional losses. More specifically, the success of Adjustment 1 is expected to be a function of *α* and *λ*, i.e. risk aversion and loss aversion. Our focus on an index insurance product means we can envisage high frequency payouts (no deductibles) due to low administrative costs as payouts are triggered automatically based on the performance of the index rather than by farm damage assessments. Moreover, moral hazard is less of an issue in the index insurance framework which reduces the need for a deductible.

In addition, the convexity of the value function in the loss domain, *∂*^2^v(*σ*)/∂*σ*^2^ ≤ 0 for *σ* < 0, implies risk seeking behavior in losses, i.e. v(−*x*) + v(−*y*) < v(−(*x* + *y*)) [30]. Translated into the narrowly framed weather insurance context, this implies that farmers prefer volatile premium payments rather than the more commonly sought stable premium payments (See note 7 of S1 Footnotes). Hence, an increase in the range of premium payments realized is likely to raise the prospect value. Moreover, in case of mixed gain/loss events, i.e. occurrence of outcome (x = insurance payout, -y = premium payment) with |*x*| < |*y*|, it is not intuitive whether v(*x*) + v(−*y*) ≷ v(*x* − *y*)). The general trend is that the smaller *x* is in relation to *y*, the more segregation of *x* and −*y* is preferred as v(*x*) + v(−*y*) > v(*x* − *y*)) tends to hold. In our weather insurance example, premium payments and insurance payouts occur jointly. When payouts are smaller than the premiums due, farmers must pay the resulting difference to the insurer so under narrow framing a loss occurs even though the insurance theoretically granted a payout. Hence, segregating gains and losses can increase the prospect value under narrow framing. Therefore, we propose a second Adjustment.

Adjustment 2: Conclude a multiyear contract and only pay premiums in years of no crop losses or, if there are no years with no losses, at the end of the contract period.

Adjustment 2, is designed to shift premium payments to a less sensitive part of the value function, with the multiyear property as precondition. If premiums are only paid every n-th year, the amount of this payment depends on how many yearly instalments are summed together. Thus, we propose to change the deterministic annual premium into a stochastic multiyear premium, which considers risk seeking behavior and declining sensitivity to losses. Moreover, farmers can postpone their premium payments in case of small insurance payouts (generated by Adjustment 1) and thus perceive these payouts as a gain. This means that the insurance contract must be extended to cover a period of several years.

#### 2.1.4. Specification of expected utility & cumulative prospect theory

The analysis is performed using different EU and CPT specifications for the empirical part. In the case of EU, we vary the measure of risk aversion ***φ*** across [**0, 0.2, 0.4, 0.6, 0.8, 1.0**]. This range is in accordance with experimentally elicited preferences of farmers in Germany (e.g. [36,37,38]). We account for the quantile risk premiums that complement the analysis on expected utility by dividing the wealth distribution into four equally large quartiles and derive the quantile risk premium based on an average ***φ* = 0.5**.

**Fig 1:**
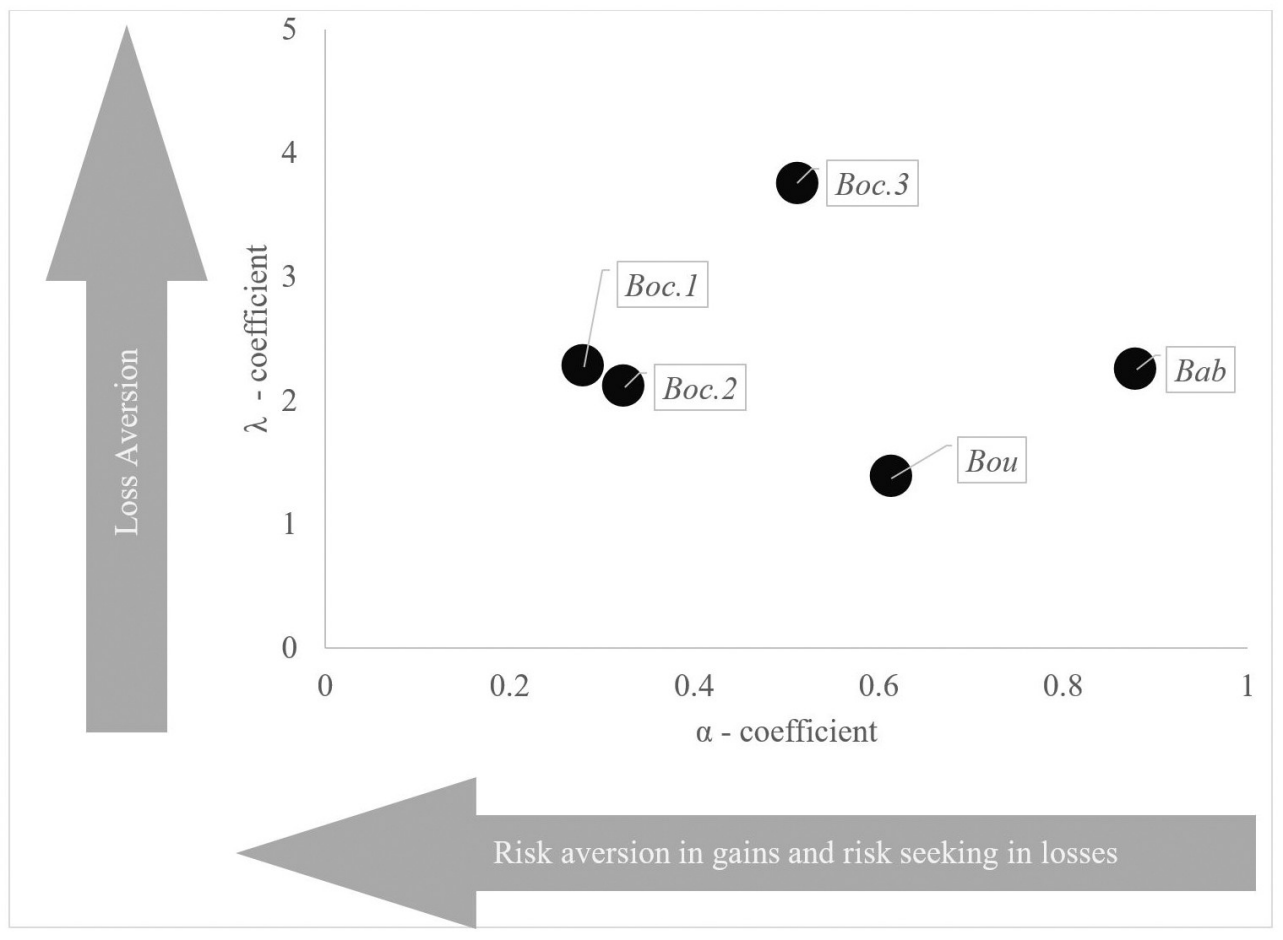
Visual classification of CPT specifications in recent studies. Flags indicate abbreviations according to Table 1.

In the case of CPT, we expect our results to be dependent on *α*, *λ* and γ/*δ*. To be exact, we use specifications from the only two empirical peer reviewed studies that have elicited CPT preferences in European agriculture [19,20]. Bocquého, Jacquet and Reynaud [19] present three sets of CPT parameters based on different estimation techniques (abbreviated as *Boc*.*1- 3* hereafter). In addition, Bougherara et al. [20] provide a fourth set of CPT elicited parameters (abbreviated as *Bou* hereafter). We use CPT specifications employed by Babcock [9], which were taken from the original cumulative prospect theory paper by Tversky & Kahneman [12] (abbreviated as *Bab* hereafter) to extend real world elicited preferences. Table 1 and Fig 1 summarize and visualize the different specifications elicited in the above papers. Here, *Boc*.*1* is characterized by a low α-coefficient indicating relatively marked risk aversion over gains and risk seeking over losses. The loss aversion coefficient λ indicates that losses are weighted almost twice as much as gains. Similarly, *Boc*.*2* implies slightly lower risk aversion in gains (and lower risk seeking in losses) and a loss aversion comparable to *Boc*.*1*. *Boc*.*3* exhibits even lower risk aversion over gains and risk seeking over losses compared to *Boc*.*1* and *Boc*.*2* but with higher loss aversion. Compared to the three scenarios above, *Bou* has lower risk aversion over gains (and risk seeking over losses) together with considerably lower loss aversion. The *Bab* specification has relatively lower risk aversion over gains and risk seeking over losses together with a loss aversion specification that is similar to *Boc*.*1* and *Boc*.*2*.

**Table 1:** CPT specifications of recent studies.

	Abbreviation	*α* − coefficient (risk aversion)^a^	*λ* − coefficient (loss aversion)	*ξ/γ/δ* − coefficient (probability distortion)^c^
Bocquého, Jacquet. and Reynaud [17]	*Boc*.*1*	0.280	2.275	0.655
	*Boc*.*2*	0.325	2.110	0.679
	*Boc*.*3*	0.512	3.756	0.647
Bougherara et al. [18]	*Bou*	0.614	1.374	0.785 / 0.844
Babcock [7]	*Bab*	0.880	2.250	0.610 / 0.690^b^

^a^ Note that smaller numbers imply higher risk aversion

^b^ According to Eq 5.1 and 5.2, different weighting functions are used for gains and losses respectively

^c^ See Eqs 4, 5.1 and 5.2 for further details of weighting functions based on Prelec [39] and Tversky & Kahneman [12]

With respect to probability weighting, all CPT specifications imply overweighting of small and underweighting of high probability values with almost similar magnitudes. The specifications employed also differ with respect to the functional forms of *ω*(*p*). Eqs 4 to 5.2 show the specifications of *ω*(*p*) as used by i) Bocquého, Jacquet and Reynaud [19] (*ω*_1_) and ii) Bougherara et al. [20] (originally proposed by Prelec [39]) and Babcock [9] ($\omega_{2}^{+}$ and $\omega_{2}^{-}$ for gain and loss probabilities respectively; originally proposed by Tversky & Kahneman, [11]):

$$\omega_{1}(p)=\text{exp}[-{\left(-\text{ln}\left(p\right)\right)}^{\xi }]$$ (4)

$$\omega_{2}^{+}(p)=\frac{p^{\gamma }}{{\left(p^{\gamma }+{\left(1-p\right)}^{\gamma }\right)}^{\frac{1}{\gamma }}}\;\omega_{2}^{-}(p)=\frac{p^{\delta }}{{\left(p^{\delta }+{\left(1-p\right)}^{\delta }\right)}^{\frac{1}{\delta }}}$$ (5.1/5.2)

We thus obtain vectors *pν*_*iαλγ*_ for the two insurance designs *κ* and the five CPT specifications. These vectors contain insurance prospect values for each farm. Taken together, the five specifications above allow us to implement a realistic range of preference scenarios that support our empirical analysis (See note 8 of S1 Footnotes [40]).

### 2.2. Testing

We investigate the proposed BWI by conducting statistical tests in three different dimensions. Firstly, we test the risk reducing properties of an actuarially fair TWI scheme against the actuarially fair BWI by comparing *eu*_*κφ*_ vectors of insured terminal wealth. We complement this by testing for changes in the quantile risk premiums Δ*R*_*kκφi*_. This allows us to pinpoint where the reduction in financial risk exposure occurs in the wealth distribution. More specifically, we test the following null hypotheses based on observations across various farms and across different levels of risk aversion *φ*:

$$\text{H}1:{\text{H}}_{0}:eu_{traditional\;\varphi }\leq eu_{no\;insurance\;\varphi }$$

$$\text{H}2:{\text{H}}_{0}:eu_{behavioral\;\varphi }\leq eu_{no\;insurance\;\varphi }$$

$$\text{H}3:{\text{H}}_{0}:eu_{behavioral\;\varphi }\leq eu_{traditional\;\varphi }$$

Secondly, we test whether the BWI scheme is better suited to farmers’ preferences (in terms of prospect value) than a TWI scheme and would be likely to increase insurance demand. Furthermore, we explore the stability of the expected performance of the BWI scheme using different CPT value function parameters. The performance of BWI and TWI is then compared across the different CPT specifications (See Table 1).

$$\text{H}4:{\text{H}}_{0}:pv_{behavioral\;boc.1}\geq pv_{traditional\;boc.1}$$

$$\text{H}5:{\text{H}}_{0}:pv_{behavioral\;boc.2}\geq pv_{traditional\;boc.2}$$

$$\text{H}6:{\text{H}}_{0}:pv_{behavioral\;boc.3}\geq pv_{traditional\;boc.3}$$

$$\text{H}7:{\text{H}}_{0}:pv_{behavioral\;bou}\geq pv_{traditional\;bou}$$

$$\text{H}8:{\text{H}}_{0}:pv_{behavioral\;bab}\geq pv_{traditional\;bab}$$

Thirdly, we clarify how the individual Adjustments that inform our approach contribute to an increase in the prospect value of the insurance. More specifically, we present results for the statistical tests of H1–H8 when BWI Adjustment 1 (“Insure small losses also—no deductible”) is revoked and when Adjustment 2 (“Conclude a multiyear contract and only pay premiums in years of no crop losses or, if there are no years with no losses, at the end of the contract period”) is revoked.

We use nonparametric paired Wilcoxon signed rank tests to compare vectors *eu*_*κφ*_ (to test for expected utility changes across levels of risk aversion), Δ*R*_*kκφi*_ (to test for quantile risk premium changes) and *pν*_*iαλγ*_ (to test for prospect value changes across levels of risk aversion in gains and risk seeking in losses, loss aversion as well as probability weighting functions). The Wilcoxon signed rank test is used to perform pairwise comparisons of the differences between the vectors stated in all the above hypotheses. We test for increases in the expected utility and prospect value and for decreases in the quantile risk premia. The Wilcoxon signed rank test calculates sample differences and ranks them based on absolute sizes. These differences are weighted by their rank and summed together. A p-value is derived from the resulting test statistic for each scenario tested.

### 2.3. Index design

**Fig 2:**
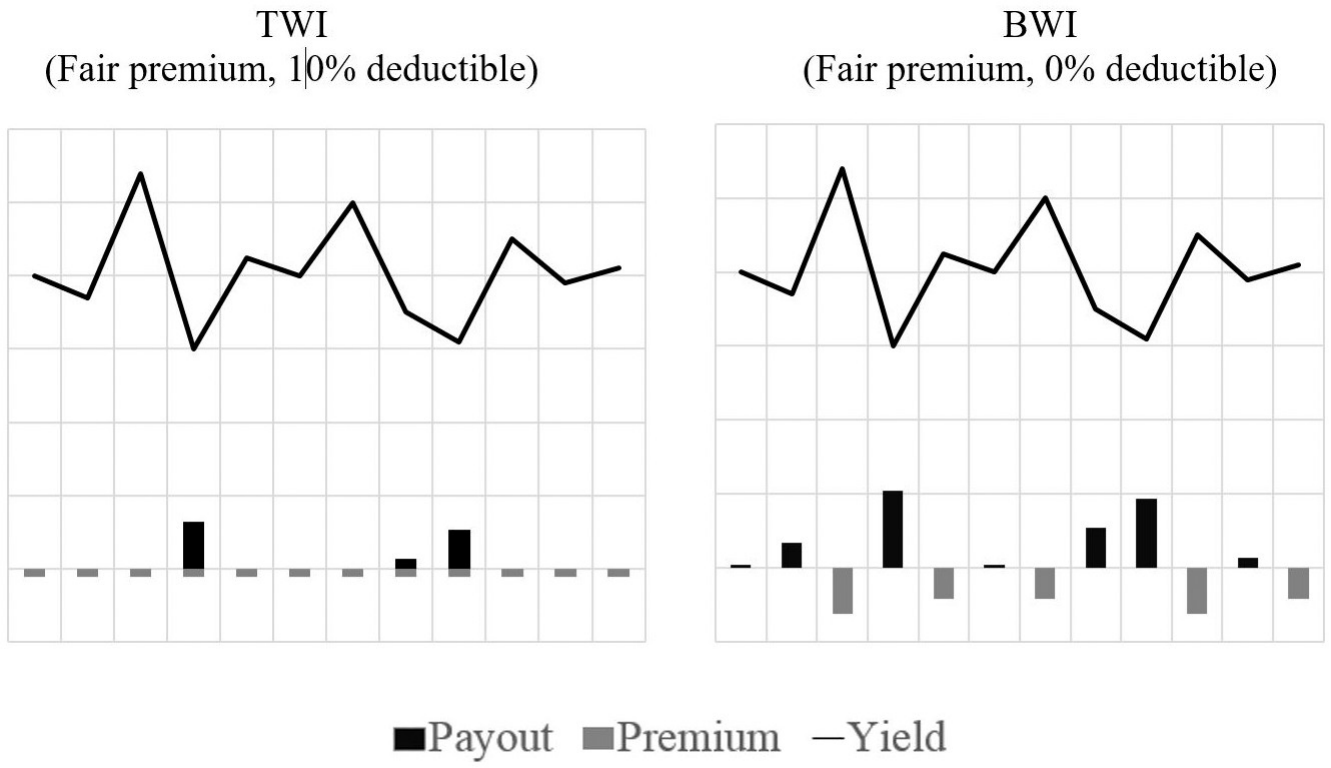
Exemplary visualization of TWI and BWI (no basis risk).

TWI and BWI both aim to provide indemnification in case of a drought event during sensitive stages of plant growth. We consider a TWI design with a standard linear payout function, a 10% deductible, and premium payment every year. TWI is compared with a BWI design that includes the two Adjustments described earlier. The multiyear contract length was fixed at three years (following Chen & Goodwin, [41]) (See note 9 of S1 Footnotes). The cumulative premium due must be paid if there is no payout at any time during the three year period. It must also be duly paid at the end of the contract period if the insurance makes payouts in each of the three years. Fig 2 illustrates an example of yields together with premiums and payouts for both TWI and BWI across a 12 year period (See note 12 of S1 Footnotes).

In accordance with Conradt et al. [42], we select the characteristics of the weather insurance contracts for each farm to minimize basis risk and maximize risk reducing properties. Thus each farm in our sample receives a farm individual insurance contract tailored to the site specific risk exposure. We focus on the coverage of drought induced yield losses in winter wheat and use a deficit of the cumulative rainfall during vulnerable plant growth stages as an indicator of drought. We therefore match farm-level yield records with historical cumulative rainfall data during the critical stages that are exogenous to our analysis (See note 11 of S1 Footnotes). We use high-resolution rainfall grid data to remove spatial basis risk [43]. Thus, the rainfall index value $r_{ti}^{R}$ for farm *i* in year *t* is calculated as the sum of rainfall $R_{d}^{ti}$ from day *d* = ‘start date’ to day *d* = ‘end date’:

$$r_{ti}^{R}=\sum_{d=start}^{end}R_{d}^{ti}$$ (6)

**Fig 3:**
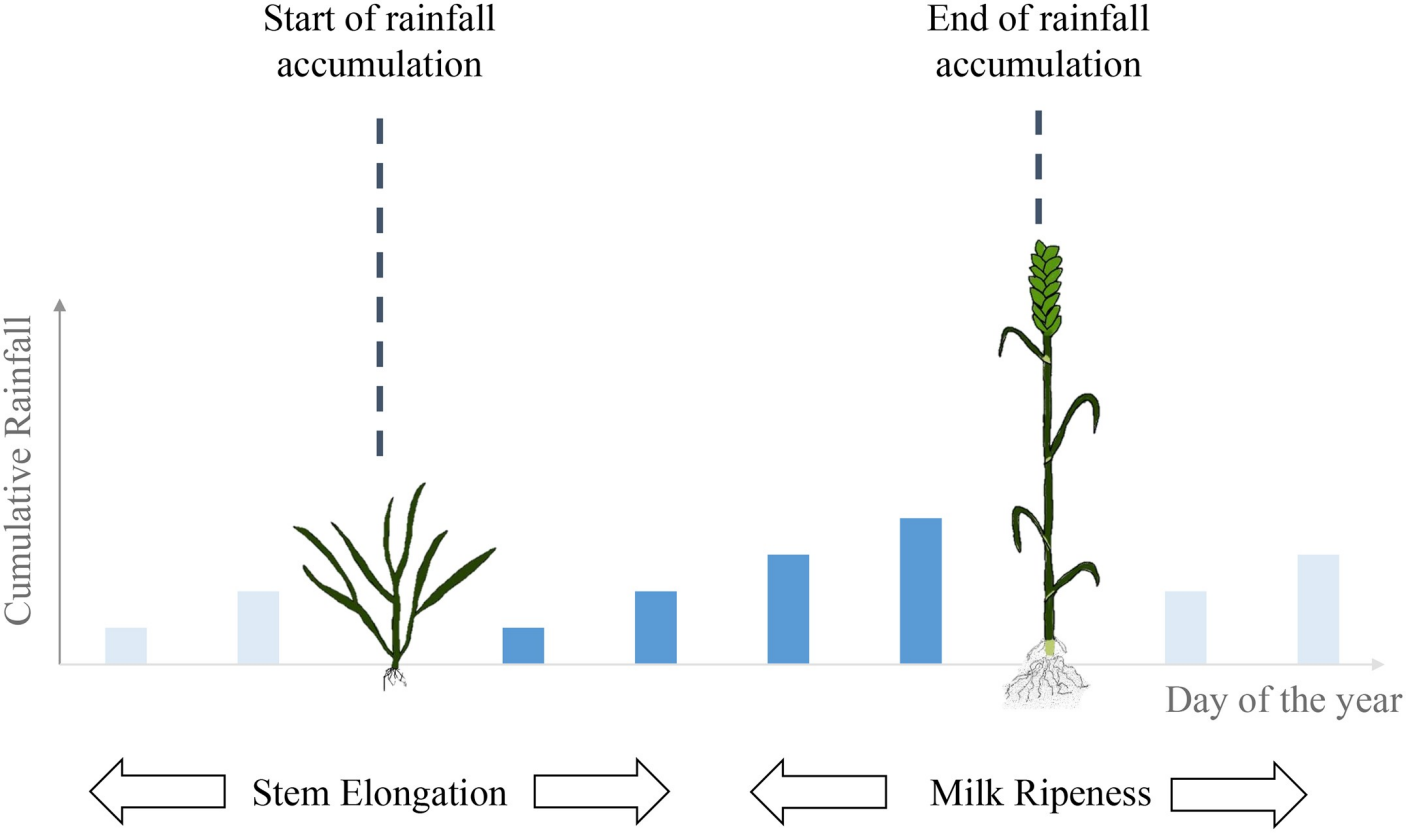
Farm individual determination of the cumulative rainfall index value using regional phenological observations to find the day of the year when stem elongation and milk ripeness occur in wheat. Rainfall is only measured between the observed phenology dates.

Start and end dates of critical plant growth stages (i.e. from winter wheat’s stem elongation to milk ripening) are determined using regional crop growth monitoring network data for each year as proposed by Dalhaus, Musshoff & Finger [44]. Thus, the farm’s individual insurance period is flexible in both space and time according to actual occurrence dates of winter wheat growth stages, which vary across reporting stations and years. See Fig 3 for a graphical illustration of the farm individual determination of $r_{ti}^{R}$ and Dalhaus [45] for further studies using this approach.

We estimate the relationship between $r_{ti}^{R}$ and farm yields *y*_*ti*_ using quantile regression that allows special emphasis to be placed on explaining certain (e.g. low) yield outcomes [42]. More specifically, we expect *y*_*ti*_ to be determined by a function $g(r_{ti}^{R})\;$ including $r_{ti}^{R}$ and other factors that are summarized within an error term *ε* and are uncorrelated with weather. The relationship between weather and yield is quantified econometrically by estimating the model

$$\;y_{i}=\alpha_{i}+{r_{i}^{R\prime }\beta }_{i}+\varepsilon_{i}$$ (7)

where ***β***_***i***_ marks the change in yields when the rainfall index value $r_{ti}^{R}$ changes by one unit (millimeter). More specifically, ***β***_***i***_ is the farm individual marginal impact of a millimeter of rainfall on a farm’s wheat yield. As we expect ***β***_***i***_ to be nonlinear across yield levels (i.e. the impact of rainfall absence is more severe when yields are low), we use quantile regression as proposed by Conradt et al. [42]. Firstly, quantile regression minimizes the absolute sum of residuals rather than the squares and secondly it allows a focus on different quantiles of the yield distribution dependent on *τ* ∈ (0, 1). More specifically, *τ* is used to weight parts of interest of the *y*_*it*_ distribution. Therefore, the estimated rainfall impact that is later used to inform the insurance payout function, is especially tailored to explain losses in yield rather than average yield levels. The minimization problem of quantile regression is

$$\beta_{i\tau }={\text{min}}_{\beta_{i\tau }\;}\sum_{t=1}^{n}\rho_{\tau }(y_{it}-r_{ti}^{R\prime }\beta_{i\tau })$$ (8)

where

$$\rho_{\tau }(y_{it}-r_{ti}^{R\prime }\beta_{i\tau })=\{\begin{array}{c} \tau |y_{it}-r_{ti}^{R\prime }\beta_{i\tau }|\;if\;y_{it}\geq r_{ti}^{R\prime }\beta_{i\tau }\\ (1-\tau )|y_{it}-r_{ti}^{R\prime }\beta_{i\tau }|\;if\;y_{it}\leq r_{ti}^{R\prime }\beta_{i\tau } \end{array}$$ (9)

We use *τ* = 0.5 to put a special emphasis on below median yield outcomes.

Since the aim of our insurance is to pay out in the event of drought, it is designed as a European put option, i.e. insurance payout $\pi_{tik}=\delta ∙[T_{ik}∙\text{max}\left\{(S_{ik}-r_{tik}^{R}),0\right\}]$. Thus, an insurance payout *π*_*tik*_ in year *t* of farm *i* and insurance contract *k* is made whenever the rainfall $r_{tik}^{R}$ falls below the farm individual strike level of rainfall *S*_*ik*_. The amount of money a farmer receives is then determined by the difference between the actual rainfall $r_{tik}^{R}$ and the strike level rainfall *S*_*ik*_ multiplied by the tick size *T*_*ik*_. The tick size indicates the payout per millimeter of deficit rainfall. The optimal tick size and strike level are determined from quantile regression results. More specifically, the strike level *S*_*ik*_ of rainfall under which a payout is triggered is estimated as the rainfall value that corresponds to the mean yield $\overset{-}{y_{i}}$ in the case of BWI, i.e. $S_{iBWI}=g^{-1}(\overset{-}{y_{i}})$, and to 90% of the mean yield in the case of TWI, i.e. $S_{ik}=g^{-1}(0.9\overset{-}{y_{i}})$. This is in accordance with Conradt et al. [42] and indemnity is not paid for below average rainfall but rather for rainfall levels that imply below average yields. Tick size *T*_*ik*_ is the estimated slope coefficient ***β***_***iτ***_.

Actuarially fair premiums for the TWI and BWI contracts are calculated using the burn rate method [46] (See note 12 of S1 Footnotes). The actuarially fair insurance premium is determined on the basis of the average payout over 10,000 bootstrapped payout realizations due to the estimated rainfall distribution. A fair premium implies that the cost of the insurance for the farmer is equal to the expected payout. In reality the insurance provider would charge a loading on the fair premium, which includes administrative costs and a profit margin. Using fair premiums enables us to focus our analysis on the differences between the two insurance contracts (TWI and BWI).

## 3. Data

In the following section we present the underlying farm level yield, crop phenology and weather datasets used.

### 3.1. Yield data

Our case study region is located in a drought prone area of eastern Germany (see Fig 3) and includes farms in the German Federal states of Mecklenburg-Western Pomerania, Brandenburg, Saxony-Anhalt, Thuringia and Saxony. Here, crop yield variability is much larger compared to other regions in Germany [47]. Our original farm-level yield (*y*_*it*_) dataset was obtained from a local insurance provider and consisted of a panel of 90 farms for the years 1995 to 2015. Each farm has a minimum size of 1,500 hectares, which is considerably higher than the German average (of 60 hectares) but representative for the eastern part of Germany. The farms are highly specialized and crop production is the main source of farm income. As a result, they have a significant interest in managing their exposure to weather risk. Farmers submitted their historical yield records for multiple crops to obtain an individual weather risk assessment from the insurance provider. We selected winter wheat as an example for this study. Based on the findings of the weather risk assessments, private non-subsidized and individually-tailored weather insurance contracts were offered to the farmers. To our knowledge, this unsubsidized weather index insurance market is a unique case in a developed country context, which underlines the importance of further improving weather insurance to help farmers to cover their weather risks (for further details see www.die-wetterversicherung.de).

Since our analysis concentrates on a single weather risk, we reduced the dataset to 38 farms that provided at least 15 years of wheat yield data and exhibited significant vulnerability to a lack of precipitation, i.e. where the estimated slope ***β***_***iτ***_ indicated a negative impact of rainfall (See note 13 of S1 Footnotes). Farms that are more vulnerable to a lack of precipitation are also more likely to be interested in a rainfall insurance. The yield data were detrended using the M-Estimator as suggested by Finger [48] [49] to account for technological trends (See note 14 of S1 Footnotes). See Table 2 for summary statistics on the detrended yield data.

**Table 2:** Summary statistics of detrended winter wheat yields in decitons (= 100kg) per hectare.

Mean	66.39
Median	66.55
Min	17.42
Max	109.88
Standard Deviation	13.90
Coefficient of Variation	0.21

### 3.2. Phenology and weather data

We use a rich phenology observation network provided by the Deutscher Wetterdienst (DWD; Engl. German Meteorological Office) [43] to define critical farm-level growth phases during which wheat is especially reactive to drought stress. As advanced by Dalhaus & Finger [43] and Dalhaus et al. [44], droughts during the periods from stem elongation to ear emergence and from ear emergence to milk ripening can be extremely detrimental to final yields. Insurance contracts include the sum of rainfall across both stages as the insured weather index $r_{ti}^{R}$ (See Fig 3 for a graphical explanation of the index building).

Rainfall grid data is used to generate records of farm-level rainfall levels for the period 1997 to 2014. More specifically, we follow Dalhaus & Finger [43] and use the RegNie weather rainfall grid, which is also provided by the DWD (available under ftp://ftp-cdc.dwd.de/). The grid is based on interpolated rain gauge data with a spatial resolution of 1km x 1km. See Rauthe et al. [50] for further details on the interpolation procedure. We used the read.regnie function supplied within the ‘esmisc’ package of the statistical software environment R to derive this weather information [51]. Using the geographical coordinates (latitude and longitude) of the farms operating site, we extracted daily rainfall information from the grid dataset and obtain a daily time series of rainfall per farm. Using the regional phenological observations we then select and sum up daily rainfall within the time period from stem elongation to milk ripeness (see S1 Fig for a graphical representation of the data manipulation procedure).

### 3.3. Data availability

Data cannot be shared publicly because the data will compromise the privacy of the human subjects. The data are available from the gvf Versicherungsmakler AG (contact via Sebastian.Mahler@gvf.de) for researchers who meet the criteria for access to confidential data.

## 4. Results

Table 3 gives a summary of statistics for the weather insurance contract parameters. The median rainfall strike level was 206.20 mm for BWI and 127.70 mm for TWI, respectively. The average tick size was 0.07 deciton/ millimeter rainfall [yield terms]. The average premium rate for the TWI was 0.6% whereas it was 2.98% for the BWI. In comparison, BWI was more expensive because it did not include a deductible. Hence the percentage of years in which payouts were made was also considerably higher in the BWI case, 58.64% compared to 15.37% for TWI on average.

**Table 3:** Summary statistics of contract parameters.

		TWI	BWI
**Strike level [millimeter]**			
Median	Across all farms	140.28	141.27
Min		4.22	16.95
Max		7560.03	584.35
**Tick size [decitons/millimeter]**			
Mean	Across all farms	0.08	
Min		0.02	
Max		0.23	
**Premium rate [in %]**			
Mean	Across all farms	0.60	2.98
Min		0.00	0.21
Max		3.49	9.04
**Years with payout [in %]**			
Mean	Across all farms	15.37	58.64
Min		5.88	17.65
Max		50.0	100.00
**Years with premium [in %]**			
Mean	Across all farms	100	0.56
Min		100	33.3
Max		100	88.2

### 4.1. Risk reducing properties of TWI and BWI according to expected utility (Step 1)

Based on the two-step procedure described above, Tables 4, 5 and 6 show Wilcoxon signed rank test results for expected utility changes in Step 1 (H1, H2 and H3). In addition, Tables 7, 8 and 9 show results for changes in quantile risk premiums when testing to identify which parts of the wealth distribution are affected by the respective insurance scenario. Test results for H1 and H2 in Table 4 reveal that both TWI and BWI significantly increase farmers’ expected utility compared to a no insurance scenario. Thus, assuming a fair premium and EU preferences, TWI and BWI products reduce weather risk and are thus beneficial for risk averse farmers. In addition, results for H3 indicate a statistically significant expected utility increase through BWI compared to TWI. This is not surprising as BWI insured larger parts of the risk at a fair premium. Results regarding hypotheses H1-H3 are robust across all the levels of risk aversion tested. No differences in expected utility are found for risk neutral decision-makers, reflecting that the premium charged is actuarially fair. In addition, Table 7 shows the Wilcoxon signed rank test results of comparing quantile risk premiums for H1-H3. Results indicate that TWI is specifically suited to reduce risk at the edges of the wealth distribution whereas BWI rather reduces risk in the second quartile, which is in line with its zero deductible design. However, while BWI is unable to insure against large losses in the first quartile, it outperforms TWI in the second, third and fourth quartile of the wealth distribution in terms of risk reduction.

**Table 4:** Wilcoxon signed rank test results for changes in expected utility (H1, H2 and H3).

Coefficient of relative risk aversion φ	p-value^a^^/^^b^		
	H1	H2	H3
	H_0_: *eu*_*traditional*_ *φ* ≤ *eu*_*no insurance*_ *φ*	H_0_: *eu*_*behavioral*_ *φ* ≤ *eu*_*no insurance*_ *φ*	H_0_: *eu*_*behavioral*_ *φ* ≤ *eu*_*traditional*_ *φ*
	H_1_: *eu*_*traditional*_ *φ* > *eu*_*no insurance*_ *φ*	H_1_: *eu*_*behavioral*_ *φ* > *eu*_*no insurance*_ *φ*	H_1_: *eu*_*behavioral*_ *φ* > *eu*_*traditional*_ *φ*
0	0.56	0.67	0.65
0.2	0.03	0.11	0.28
0.4	1.97 · 10^−3^	0.02	0.08
0.6	7.27 · 10^−4^	0.01	0.04
0.8	7.27 · 10^−4^	0.02	0.03
1.0	7.27 · 10^−4^	0.01	0.03

^a^ Low p-values imply a rejection of the null hypotheses stated in H1-H3

^b^ Bonferroni corrected p-values

**Table 5:** Wilcoxon signed rank test results for changes in expected utility when Adjustment 1 is revoked (insure small losses also) (H1, H2 and H3).

Coefficient of relative risk aversion φ	p-value^a^^/^^b^		
	H1	H2	H3
	H_0_: *eu*_*traditional*_ *φ* ≤ *eu*_*no insurance*_ *φ*	H_0_: *eu*_*behavioral*_ *φ* ≤ *eu*_*no insurance*_ *φ*	H_0_: *eu*_*behavioral*_ *φ* ≤ *eu*_*traditional*_ *φ*
	H_1_: *eu*_*traditional*_ *φ* > *eu*_*no insurance*_ *φ*	H_1_: *eu*_*behavioral*_ *φ* > *eu*_*no insurance*_ *φ*	H_1_: *eu*_*behavioral*_ *φ* > *eu*_*traditional*_ *φ*
0	0.56	0.38	0.48
0.2	0.03	0.03	0.16
0.4	1.97 · 10^−3^	1.97 · 10^−3^	0.15
0.6	7.27 · 10^−4^	1.62 · 10^−3^	0.08
0.8	7.27 · 10^−4^	2.37 · 10^−3^	0.08
1.0	7.27 · 10^−4^	4.13 · 10^−3^	0.09

^a^ Low p-values imply a rejection of the null hypotheses stated in H1-H3

^b^ Bonferroni corrected p-values

**Table 6:** Wilcoxon signed rank test results for changes in expected utility when Adjustment 2 (Conclude a multiyear contract and only pay premiums in years of no crop losses or, if there are no years with no losses, at the end of the contract period) is revoked (H1, H2 and H3).

Coefficient of relative risk aversion φ	p-value^a^^/^^b^		
	H1	H2	H3
	H_0_: *eu*_*traditional*_ *φ* ≤ *eu*_*no insurance*_ *φ*	H_0_: *eu*_*behavioral*_ *φ* ≤ *eu*_*no insurance*_ *φ*	H_0_: *eu*_*behavioral*_ *φ* ≤ *eu*_*traditional*_ *φ*
	H_0_: *eu*_*traditional*_ *φ* > *eu*_*no insurance*_ *φ*	H_1_: *eu*_*behavioral*_ *φ* > *eu*_*no insurance*_ *φ*	H_1_: *eu*_*behavioral*_ *φ* > *eu*_*traditional*_ *φ*
0	0.56	0.67	0.66
0.2	0.03	0.03	0.04
0.4	1.97 · 10^−3^	2.31 · 10^−3^	3.19 · 10^−3^
0.6	7.27 · 10^−4^	3.48 · 10^−4^	1.36 · 10^−3^
0.8	7.27 · 10^−4^	2.79 · 10^−4^	5.04 · 10^−4^
1.0	7.27 · 10^−4^	2.38 · 10^−4^	4.36 · 10^−4^

^a^ Low p-values imply a rejection of the null hypotheses stated in H1-H3

^b^ Bonferroni corrected p-values

**Table 7:** Wilcoxon signed rank test results for changes in quantile risk premiums with a coefficient of relative risk aversion φ = 0.5 –all adjustments fulfilled.

		p-value^a^^/^^b^			
		Q1^c^ (0.25)	Q2 (0.5)	Q3 (0.75)	Q4 (1)
H1	H_0_: *rp*_*traditionalφ*_ ≥ *rp*_*no insuranceφ*_	0.06	0.44	0.09	7.30 · 10^−4^
	H_1_: *rp*_*traditionalφ*_ < *rp*_*no insuranceφ*_				
H2	H_0_: *rp*_*behavioralφ*_ ≥ *rp*_*no insuranceφ*_	0.23	0.01	0.27	2.16 · 10^−3^
	H_1_: *rp*_*behavioralφ*_ < *rp*_*no insuranceφ*_				
H3	H_0_: *rp*_*behavioralφ*_ ≥ *rp*_*traditionalφ*_	0.33	7.05 · 10^−3^	5.90 · 10^−3^	3.61 · 10^−3^
	H_1_: *rp*_*behavioralφ*_ < *rp*_*traditionalφ*_				

^a^ Low p-values imply a rejection of the null hypotheses stated in H1-H3

^b^ Bonferroni corrected p-values

^c^ Values in brackets indicate the upper probability bound of the respective quantile

**Table 8:** Wilcoxon signed rank test results for changes in quantile risk premiums with a coefficient of relative risk aversion φ = 0.5—when Adjustment 1 (insure small losses also) is evoked.

		p-value^a^^/^^b^			
		Q1^c^ (0.25)	Q2 (0.5)	Q3 (0.75)	Q4 (1)
H1	H_0_: *rp*_*traditionalφ*_ ≥ *rp*_*no insuranceφ*_	0.06	0.44	0.09	7.30 · 10^−4^
	H_1_: *rp*_*traditionalφ*_ < *rp*_*no insuranceφ*_				
H2	H_0_: *rp*_*behavioralφ*_ ≥ *rp*_*no insuranceφ*_	0.05	0.80	0.02	7.30 · 10^−4^
	H_1_: *rp*_*behavioralφ*_ < *rp*_*no insuranceφ*_				
H3	H_0_: *rp*_*behavioralφ*_ ≥ *rp*_*traditionalφ*_	0.03	0.98	0.67	0.29
	H_1_: *rp*_*behavioralφ*_ < *rp*_*traditionalφ*_				

^a^ Low p-values imply a rejection of the null hypotheses stated in H1-H3

^b^ Bonferroni corrected p-values

^c^ Values in brackets indicate the upper probability bound of the respective quantile

**Table 9:** Wilcoxon signed rank test results for changes in quantile risk premiums with a coefficient of relative risk aversion φ = 0.5—when Adjustment 2 (Conclude a multiyear contract and only pay premiums in years of no crop losses or, if there are no years with no losses, at the end of the contract period) is revoked.

		p-value^a^^/^^b^			
		Q1^c^ (0.25)	Q2 (0.5)	Q3 (0.75)	Q4 (1)
H1	H_0_: *rp*_*traditionalφ*_ ≥ *rp*_*no insuranceφ*_	0.06	0.44	0.09	7.30 · 10^−4^
	H_1_: *rp*_*traditionalφ*_ < *rp*_*no insuranceφ*_				
H2	H_0_: *rp*_*behavioralφ*_ ≥ *rp*_*no insuranceφ*_	8.89 · 10^−3^	0.02	0.39	1.20 · 10^−4^
	H_1_: *rp*_*behavioralφ*_ < *rp*_*no insuranceφ*_				
H3	H_0_: *rp*_*behavioralφ*_ ≥ *rp*_*traditionalφ*_	0.01	0.02	0.37	1.90 · 10^−4^
	H_1_: *rp*_*behavioralφ*_ < *rp*_*traditionalφ*_				

^a^ Low p-values imply a rejection of the null hypotheses stated in H1-H3

^b^ Bonferroni corrected p-values

^c^ Values in brackets indicate the upper probability bound of the respective quantile

In addition to Table 4, where both Adjustments of BWI are fulfilled, Table 5 shows tests for expected utility changes when Adjustment 1 is revoked. Here, BWI only differs from TWI with respect to the stochastic multiyear premium (Adjustment 2), i.e. small losses are uninsured. Accordingly, results for H1 do not differ from those displayed in Table 4. Furthermore, results for H2 show that expected utility of BWI is significantly greater compared to a no insurance scenario. Hence, assuming a fair premium, BWI without Adjustment 1 can significantly reduce farmers’ financial exposure to weather risk. To be precise, H3 reveals that no differences exist between TWI and BWI at the 5% significance level with respect to expected utility changes. This was no surprise as the Adjustments made were specifically suited to CPT decision-makers. In addition, results in Table 8 show that BWI can no longer reduce financial exposure to weather risk in the second quartile of the wealth distribution. In contrast, it now reduces risk in the first quartile.

Table 6 shows test results for H1-H3 when Adjustment 1 is reactivated and Adjustment 2 is revoked. Here, small losses are insured and premiums are due every year. Results for H1 are in accordance to those of Tables 4 and 5. Moreover, results for H2 show that BWI insurance for small losses can significantly increase expected utility and thus reduce farmers’ financial exposure to weather risk. Based on the above results, it is not surprising that H3 shows that expected utility of BWI for the farmers insured is higher than with TWI as more risk is covered at a fair rate. In addition, Table 9 suggests that with this specification, BWI reduces risk in both the first and the second quantile of the wealth distribution.

### 4.2. Prospect value changes of TWI and BWI according to cumulative prospect theory (Step 2)

The previous section focused on comparing TWI and BWI presupposing that farmers’ preferences are characterized by standard EU assumptions. This section makes the same comparison but under the assumption that farmers’ preferences are characterized by CPT instead. Based on Step 2 of our analysis, i.e. investigation of the performance of the different insurance schemes across CPT specifications, Table 10 shows significance levels of Wilcoxon signed rank test results of hypotheses H4 to H8, associated with three different BWI designs, i.e. all Adjustments fulfilled, Adjustment 1 revoked, Adjustment 2 revoked.

**Table 10:** Wilcoxon signed rank test results for differences in the prospect value using CPT specifications *Boc*.*1*, *Boc*.*2 Boc*.*3* and *Bab* (H4 –H8).

	BWI with all Adjustments fulfilled	BWI excluding Adjustment 1 (small losses not insured)	BWI excluding Adjustment 2 (payment every year)
Specifications	p-value^a^^,^^b^		
	H_0_: *pv*_*behavioral*_ ≤ *pv*_*traditional*_		
	H_1_: *pv*_*behavioral*_ > *pv*_*traditional*_		
*H4*: *Boc*.*1*	0.99	1.22 · 10^−4^	0.99
*H5*: *Boc*.*2*	1	3.05 · 10^−4^	1
*H6*: *Boc*.*3*	1	1.22 · 10^−4^	1
*H7*: *Bou*	1	0.20	1
*H8*: *Bab*	1	1.22 · 10^−4^	1

^a^ Low p-values imply a rejection of the null hypotheses stated in H4-H8

^b^ Bonferroni corrected p-values

**Fig 4:**
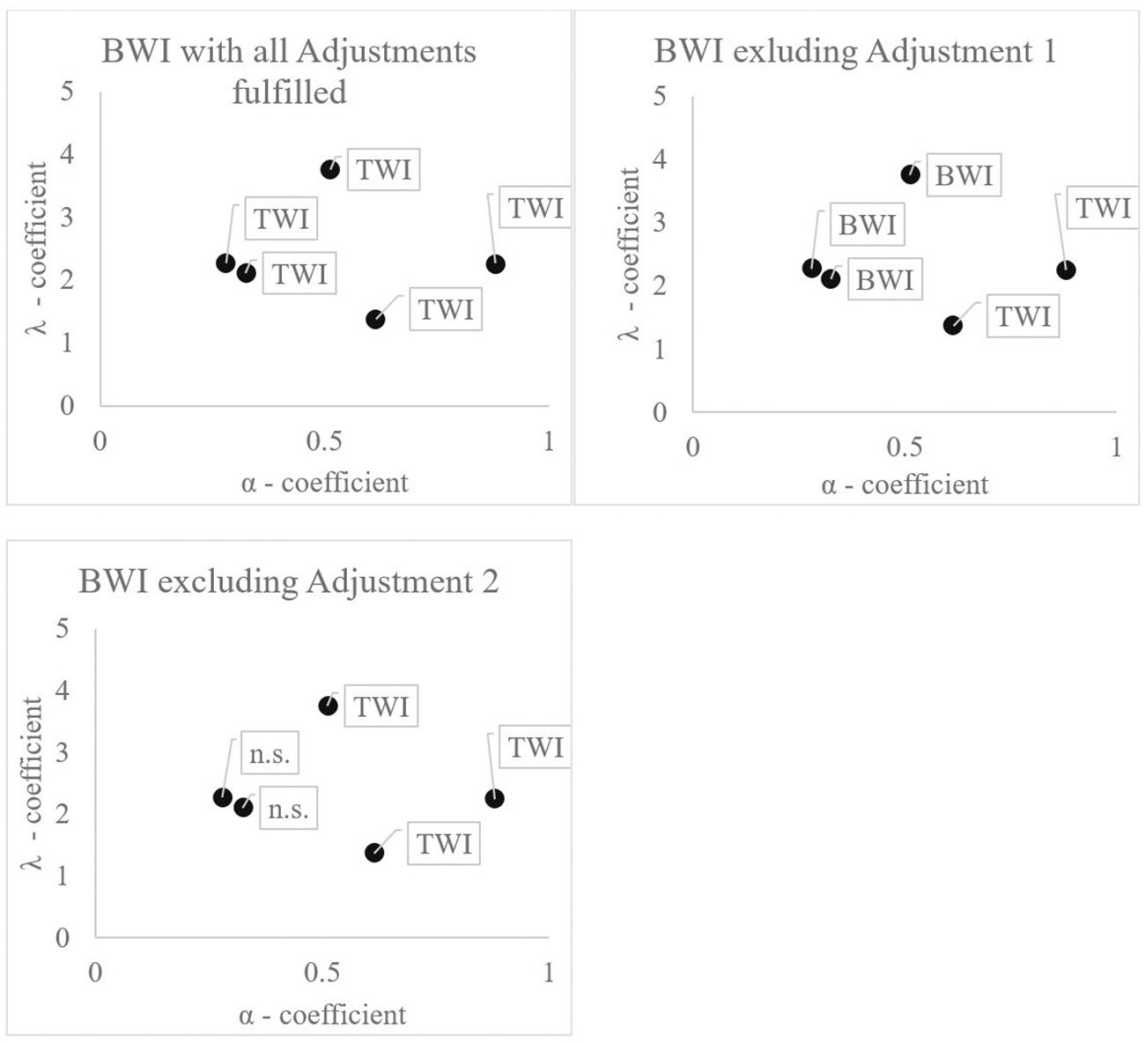
Which weather insurance scheme is preferred in which CPT specification.

The second column of Table 10 and the upper left graph of Fig 4 present BWI results with all Adjustments described above in force (according to Table 4 of Section 4.1). In this case, BWI provides no significant improvement in prospect value when compared to TWI for all CPT specifications, i.e. Bocquého, Jacquet and Reynaud [19] (*Boc*.*1*, *Boc*.*2* and *Boc*.3), Bougherara et al. [20] (*Bou*) and Babcock [9] (*Bab*).

The third column of Table 10 shows results for BWI excluding Adjustment 1 (“Insure small losses also”). Contrary to the first specification, BWI outperformed (in terms of prospect value) TWI for specifications *Boc*.*1*, *Boc*.*2*, *Boc*.3 and *Bab*. Compared to the preceding column, this BWI design introduces greater uncertainty in the loss domain, while overall losses are smaller due to lower premiums resulting from the implementation of a deductible. Thus, the effect of economies in premium payments outweighs the effect of covering small losses for these specifications. It could be said that loss aversion (the premium payments would be small losses for the insured party) clearly dominates risk aversion in gains in these cases.

The fourth column of Table 10 shows results for BWI excluding Adjustment 2 (“Conclude a multiyear contract and only pay premiums in years of no crop losses or, if there are no years with no losses, at the end of the contract period”). Here, TWI outperforms BWI (in terms of prospect value) across all specifications. In this scenario, premium payments (small losses from the perspective of the insured party) come every year and the overall amount of losses is experienced more intensively as single year premium payments appear close to the reference point (See note 15 of S1 Footnotes).

n.s. = not significant. No insurance outperformed the other.

Note: Flags indicate the outperforming insurance scenario according to test results displayed in Table 3. The underlying literature sources of CPT preferences can be derived from Fig 1.

## 5. Discussion

Babcock [9] shows that loss aversion can lead to a reduction in optimal crop insurance coverage levels and thus to less protection against potential income losses, when farmers narrowly frame their insurance as a stand-alone investment rather than a risk management tool. The BWI proposed here aims to counteract this tendency by accounting for CPT properties of farmers’ preferences under this narrow framing. This includes transforming single year premiums into multiyear premiums (Adjustment 2). Since there is no deductible (Adjustment 1), a farmer can also experience frequent small gains (insurance payouts) with BWI. We introduce a two-step procedure to test the Adjustments with respect to changes in the risk reducing properties (Step 1: Expected Utility Theory) and changes in the prospect value (Step 2: Cumulative Prospect Theory) under various real world preference scenarios.

In the case of Step 1, we find that the actuarially fair BWI objectively reduces weather risk exposure as each of the Adjustments separately, and the combination of both Adjustments, increase EU. Our quantile risk premium analysis reveals a weak statistical significance for changes in the risk at lower quartiles for TWI. Clarke [52] suggests that weather insurance becomes unattractive for risk averse decision-makers if it worsens bad financial situations, i.e. in case of basis risk [53]. Thus, it is still important to further reduce the basis risk, i.e. the discrepancy between insurance payout and financial losses on the farm. Most recently, Lampe & Würtenberger [13] suggested a model for farmers that incorporate basis risk in their framing of the insurance product. Using their findings to design a behavioral weather insurance also for these farmers can further increase the attractiveness of index insurances.

Step 2 reveals that even with both Adjustments implemented jointly, BWI is still unable to increase the prospect value of farmers that narrowly frame insurance as a stand-alone investment when compared to TWI. However, when Adjustment 1 is revoked, BWI increases the prospect value compared to TWI for CPT specifications in *Boc*.*1*, *Boc*.*2*, *Boc*.*3* and *Bab*. Therefore, higher loss aversion, as observed in these specifications, increases preferences for BWI compared to TWI. This is due to the fact that when Adjustment1 is implemented alone, BWI entails stochastic multiyear premiums instead of the deterministic yearly premiums payable with TWI. Hence, losses occur further away from the reference point. Therefore, stochastic multiyear premiums potentially increase the insurance demand of prospect value maximizing farmers that narrowly frame insurance as a stand-alone investment. It must be noted that postponing premium payments can also open the way to strategic default and action may have to be taken to avoid this behavior [54]. Thus, BWI requires a strong institutional environment with a strong legal system to enforce the insurance contract.

In contrast, a zero deductible design (Adjustment 1) does not benefit farmers in terms of prospect value under the narrow framing assumption as it increases the total amount of premium payments which are framed as losses in the CPT setting. This persists in the situation when both Adjustments are fulfilled and also when only Adjustment 1 is implemented. This result is in line with Babcock [7] who shows that prospect value maximizing farmers with relatively low risk aversion and average loss aversion prefer insurance with higher deductibles. Consequently, while a stochastic multiyear premium is in itself prospect value increasing, it is nevertheless not able to counteract the overweighting of premium payments framed as losses. Thus the ‘segregation of silver linings’ is unable to counterbalance loss aversion in our case study. In this context, Du, Feng & Hennessy [6] show that farmers are reluctant to buy actuarially fair insurance as out of pocket premium payments increase. This underlines that farmers avoid higher premium payments, even if these are fair and objectively reflect their risk profile. While our findings suggest that farmers have little incentive to insure small/ moderate losses under CPT narrow framing [9,10,11], Sydnor [14] detects over-insurance of modest risks (i.e. the choice of low deductibles) in home insurance markets. He suggests potential explanations such as, biased subjective beliefs, differences between global and local utility, extreme liquidity constraints. Hence, these drivers might also influence the deductible choice made by farmers in agricultural insurance settings. This could be tested in future research. Moreover, various studies detect an increase in farmers’ insurance demands after experiencing a payout or a loss [55,56,57,58]. Obviously, this cannot be explained by the narrow framed decision-making under CPT used here and further research is needed to shed more light on behavioral crop insurance demand. Numerous economic experiments that study insurance demand provide a rich source of information on the role of various preferences on the demand for different insurance types (see Jaspersen, [59] for an overview). The application of these experiments in an agricultural insurance setting is a logical continuation of past research. However, there is a notable lack of investigation into the effect of framing or communication (e.g. [60]), social factors [61] or emotions [62] on insurance demand in agriculture and the moral hazard factor has hitherto also been neglected. A better understanding of the connections between these characteristics and decisions might improve the uptake of insurance as well as its environmental consequences. To summarize, additional knowledge about the driving characteristics of farmers’ insurance demands and deductible choices could provide the foundation for an additional theoretical model that might be the underlying driver. One entry point can be the work of Scholten & Read [63] who integrate Markowitz’ [64] “forgotten fourfold pattern of risk preferences” into cumulative prospect theory by allowing switches between risk aversion to risk seeking dependent on the size of the outcome (gain or loss) that is at stake. In addition to the cumulative prospect theory, the salience theory [65] could also represent another interesting course to follow. Here, decision-makers are driven by their attraction to a specific outcome rather than their aversion.

It should be noted that our test results rely on the framing of insurance as a stand-alone investment and this dictates the choice of the reference point. As stated, we expect that there is a wide range of decision-making behavior among farmers and in particular differences in the reference point may necessitate further Adjustments. Therefore, we regard our analysis as first step towards a specific tailoring of insurance to align it with farmers’ decision-making behavior. In particular, the findings of Kőszegi & Rabin [66], Schmidt, Starmer & Sugden [67] and Schmidt [68] offer various points of departure for adjusting the reference point to include basis risk and frame it as a loss. Feng, Du & Hennessy [7] take up the findings of Schmidt, Starmer & Sugden [67] and test for state dependent reference points in hypothetical crop insurance decisions among US farmers. Their findings imply that based on state-dependent reference levels “a strong distaste for paying premium” can be explained. Future research must take this into account and experimental findings on farmers’ behavior should be included in insurance design. Elabed & Carter [69] provide further behavioral economic insights into how compound risk aversion can influence index insurance demand under basis risk. This offers another point of departure for ongoing research. In addition, Sung et al. [17] propose an optimal behavioral insurance under the prospect theory, where the whole wealth distribution is considered in the reference point. In light of the extensive literature already available on optimal crop insurance under expected utility, this seems to be a logical next step [15,16]. Moreover, Lampe & Würtenberger [13] find that a narrow stand-alone investment framing of index insurance contracts can be related to a low understanding of the index insurance product, i.e. the reference level is state dependent. This suggests that the behavioral weather insurance proposed here could be especially suited to incentivize the demand for index insurance among farmers that have a limited understanding of the insurance contract. However, as Lampe & Würtenberger [13] focus on a case study in India their findings on farmers’ preferences might only be partly applicable to our central European case-study due to large differences between the two agricultural systems (see also Petraud, Boucher & Carter [70]).

As our results rely on simulations, their viability must be proved in the field before we can offer a BWI in line with market requirements. Furthermore, it is important to note that the analysis presented here is based on data from large farms in eastern Germany. Given differences in institutional structures and possible divergences in risk preferences, future research could examine whether or not these findings can be applied to developing country contexts where considerable efforts are currently being made to develop weather insurance markets [71].

## 6. Conclusion

To summarize, our approach proposes at two-step procedure to develop an insurance product that has risk reducing properties under expected utility and to evaluate the prospect value of insurance under CPT. We test for various preference scenarios under both theories. Our strategy involves a temporal redistribution of money flows to frame crop insurance in a way that we believe may be more attractive to farmers. We find that BWI is preferable to TWI for farmers with expected utility preferences. This was to be expected given its zero deductible design at a fair premium. In addition, when either of the two Adjustments is revoked, BWI still reduces the financial exposure to weather risk and is preferred by farmers with expected utility preferences. Moreover, and most importantly, depending on the assumed CPT preference specifications, farmers with CPT preferences may prefer BWI over TWI if the stochastic multiyear premium is implemented (Adjustment 2) alone. BWI could thus lead to an increase of insurance demand.

This leads to certain significant conclusions. Firstly, to our knowledge this is the first study explicitly designing crop insurance in general and weather insurance in particular, on the basis of farmers’ CPT preferences. In this way, we are able to show how integrated premium payments combined with a multiyear contract, can lead to an increase in insurance demand. Secondly, we show that the relative benefits of the BWI depend strongly on assumed CPT value function characteristics, such as the degree of loss aversion. This means that farmers’ characteristics must be considered when designing individual crop insurance contracts with a view to increasing the attractiveness of the contracts and thus encourage insurance purchases. Hence, it is worthwhile to consider offering multiple types of contracts. Thirdly, potentially more farmers are insured against downside risks with BWI compared to the current state. This makes the farming system as a whole more resilient against climate shocks. Finally, we tested across a wide range of risk scenarios (as displayed through the very heterogeneous premium rates) using the contract parameters shown in Table 3 and weather summary statistics in Table 11. This leads us to conclude that our findings can be upscaled to meet other hazards in other regions.

**Table 11:** Summary statistics of cumulative rainfall from stem elongation to milk ripening in liters per square meter.

Mean	133.73
Median	127.1
Min	9.1
Max	402.7
Standard Deviation	56.14
Coefficient of Variation	0.42

Future research should continue to focus on farmers’ insurance decision-making and, in particular, should investigate the role of the framing situation and possible contract adjustments. In particular, state-dependent framing and reference levels seem to offer promise for better explaining farmers’ insurance demand. The framework proposed here should not be seen as a static product but rather as an entry point for the dynamic development of new behavioral considerations in crop insurance contract design. Future research on behavioral insurance design must take up future findings on farmers’ insurance decision making to optimally suit farmers’ preferences. There are various links to literature on experimental derivation of insurance decisions, optimal behavioral insurance design outside agriculture and promising concepts such as salience theory, which should be helpful in identifying a methodological starting point.

## Supporting information

S1 Data (DOCX)

S1 Fig Data manipulation procedure to obtain the rainfall index that is used as insurance underlying. For the last step see Fig 3 in the main body of the paper. The procedure is also described in section *2*.*3 Index design* in the main body of the paper.
(DOCX)

S1 Footnotes (DOCX)

S1 Formula Incremental risk premium. (DOCX)

S1 Table Wilcoxon signed rank test results for changes in expected utility (H1, H2 and H3)–sensitivity analysis two-year contract. (DOCX)

S2 Table Wilcoxon signed rank test results for changes in expected utility (H1, H2 and H3)–sensitivity analysis four-year contract. (DOCX)

S3 Table Wilcoxon signed rank test results for changes in expected utility when Adjustment 1 (insure small losses also) (H1, H2 and H3) is revoked—sensitivity analysis two-year contract. (DOCX)

S4 Table Wilcoxon signed rank test results for changes in expected utility when Adjustment 1 (insure also small losses) (H1, H2 and H3) is revoked—sensitivity analysis four-year contract. (DOCX)

S5 Table Wilcoxon signed rank test results for differences in the prospect value using CPT specifications *Boc*.*1*, *Boc*.*2 Boc*.*3* and *Bab* (H4 –H8)–sensitivity analysis two-year contract. (DOCX)

S6 Table Wilcoxon signed rank test results for differences in the prospect value using CPT specifications *Boc*.*1*, *Boc*.*2 Boc*.*3* and *Bab* (H4 –H8)–sensitivity analysis four-year contract. (DOCX)

S7 Table Wilcoxon signed rank test results for differences in the prospect value using CPT specifications *Boc*.*1*, *Boc*.*2 Boc*.*3* and *Bab* (H4 –H8)—sensitivity analysis 20% loading. (DOCX)

S8 Table Wilcoxon signed rank test results for differences in the prospect value using CPT specifications *Boc*.*1*, *Boc*.*2 Boc*.*3* and *Bab* (H4 –H8)—sensitivity analysis discounting multiyear premiums and pay outs to the year of contract closure (2% interest rate). (DOCX)
