# Supplementary material for: Behavioral weather insurance: Applying cumulative prospect theory to agricultural insurance design under narrow framing
Source: PLoS One. 2020 May 1;15(5):e0232267. doi: 10.1371/journal.pone.0232267 (PMC7194365; doi:10.1371/journal.pone.0232267)
Supplement: S1 Data — (DOCX) [file pone.0232267.s001.docx]

**S10 Literature Background on the behavioral economics of insurance decisions.**

Numerous studies have shed light on the impact of decision makers’ preferences on their insurance demand. These theories offer interesting opportunities for future research to test whether crop insurance demand can possibly be better explained. For instance, Friedl, Miranda & Schmidt (2014) show that social comparison leads to reduced insurance demand for disaster insurance through social reference points. Thus, interactions between neighboring farmers might play a role in the crop insurance decision.

Moreover, Jindal (2015) develops a model to explain extended warranty purchase in washing machines, a form of over-insurance. This might be used by future research to explain over-insurance in agriculture in case this is observed. Regarding this, Meeker et al. (2015) show how a small loss insurance can be seen as an incentive strategy. A concept that has proven not to work in our crop insurance application.

Mattos & Zinn (2016) show that an agricultural producer’s reference price is non-stable and determined by “the current market price, the highest price to date, and their expectation about price behavior”. This research can support future research to find reference levels of a CPT framework that helps to better explain crop insurance decisions.

Finally, Sproul, Zilberman & Cooper (2013) show that expected utility theory can be used to assess shallow-loss and coinsurance policies for US farmers.

**References**

Friedl, A., De Miranda, K. L., & Schmidt, U. (2014). Insurance demand and social comparison: An experimental analysis. *Journal of Risk and Uncertainty*, 48(2), 97-109.

Jindal, P. (2015). Risk preferences and demand drivers of extended warranties. *Marketing Science*, *34*(1), 39-58.

Mattos, F. L., & Zinn, J. (2016). Formation and adaptation of reference prices in grain marketing: an experimental study. *Agricultural economics*, *47*(6), 621-632.

Meeker, D., Thompson, C., Strylewicz, G., Knight, T. K., & Doctor, J. N. (2015). Use of Insurance Against a Small Loss as an Incentive Strategy. *Decision Analysis*, *12*(3), 122-129.

Sproul, T.W., D. Zilberman and J.C. Cooper, (2013). Deductibles vs. coinsurance in shallow-loss crop insurance. Choices , 28(3), pp. 1-4.
