# Supplementary material for: Behavioral weather insurance: Applying cumulative prospect theory to agricultural insurance design under narrow framing
Source: PLoS One. 2020 May 1;15(5):e0232267. doi: 10.1371/journal.pone.0232267 (PMC7194365; doi:10.1371/journal.pone.0232267)
Supplement: S1 Fig — For the last step see Fig 3 in the main body of the paper. The procedure is also described in section 2.3 Index design in the main body of the paper. (DOCX) [file pone.0232267.s002.docx]

**S11 Procedure to obtain rainfall grid data**


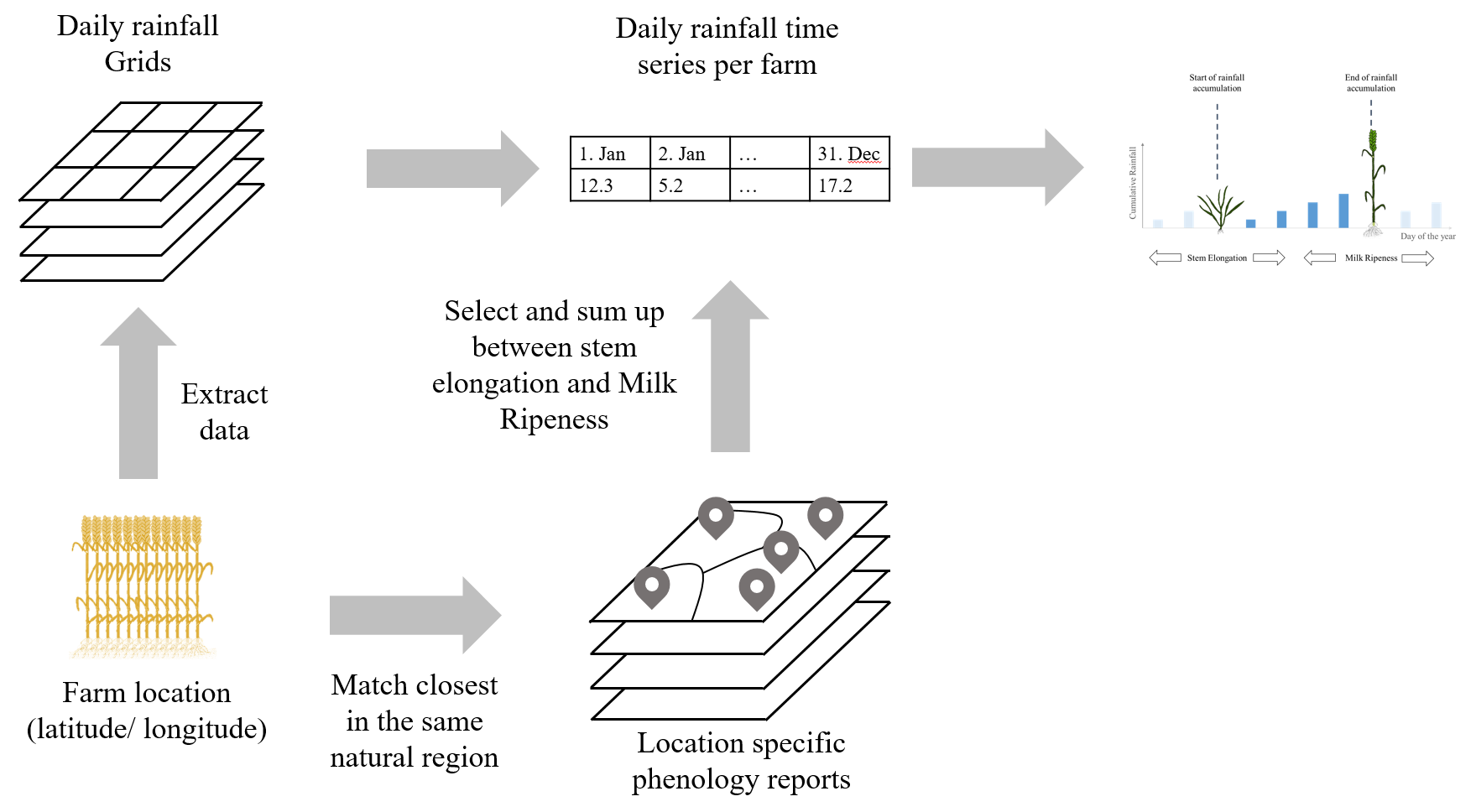


**Figure S11: Data manipulation procedure to obtain the rainfall index that is used as insurance underlying. For the last step see Fig 3 in the main body of the paper. The procedure is also described in section *2.3 Index design* in the main body of the paper.**
