# Supplementary material for: Behavioral weather insurance: Applying cumulative prospect theory to agricultural insurance design under narrow framing
Source: PLoS One. 2020 May 1;15(5):e0232267. doi: 10.1371/journal.pone.0232267 (PMC7194365; doi:10.1371/journal.pone.0232267)
Supplement: S1 Footnotes — (DOCX) [file pone.0232267.s003.docx]

**S 12 Footnotes**

A small number of countries with functioning non-subsidized crop insurance markets, such as Switzerland [70], represent exceptions and do not preclude this general tendency.

2 We are aware that our assumption (based on Babcock, [8]) that farmers view insurance as a stand-alone investment can be interpreted to contradict the findings of Tversky & Kahneman [11] who argue that the weighting characteristic of CPT is the more probable explanation behind an insurance decision. In fact, the framework we present here also allows the insurance design to include other decision-making processes that might exist within the farming community. We hope that our findings encourage future research to provide more experimental evidence on farmers’ insurance decisions. In particular, this should extend beyond a solely CPT focused approach, so that it can be also considered in insurance design.

3 The insurance does not protect against stochastic prices and we do not expect a natural hedge at the farm-level. Thus, for the analysis presented here, prices are excluded and terminal wealth is used in yield units.

4 Note that α is an anti-index for risk aversion. 0 <$\alpha$< 1 implies risk aversion, $\alpha$ = 1 risk neutrality, and $\alpha$ > 1 risk seeking in gains and the respective opposite in losses.

5 Barsefhyan, Prince & Teitelbaum [31], even find intra-subject differences of preferences across risky domains. An extension of our research to investigate differences across insurance domains (e.g. crops) appears to be a promising topic for upcoming studies.

6 In fact, recent studies by Lampe and Würtenberger [12] and Feng, Du & Hennessy [7] suggest that farmers frame insurance differently among different states of nature. Designing behavioral weather insurance for state dependent reference levels appears to be an interesting topic for future research. The behavioral weather insurance proposed here is aimed at those farmers and states in which the insurance decision is narrowly framed as a stand-alone investment.

7 In fact, year-to-year differences can occur depending on past loss experiences. However, these mostly constitute minor changes in the premium.

8 It is a point of discussion in applied economics research whether farmers’ risk preferences are stable over time or whether they change based on past experience. For the interested reader we point towards Carter [71]. As we are unable to observe farmers actual preferences over time, we acknowledge this by testing our results based on a set of different preferences, each of which is stable over time.

^9^ Our results were robust against changes in the contract length. See the online supplementary file for results using contracts covering two and four years.

^10^ Our results were qualitatively robust against the consideration of discounting. We discounted all payouts and premium payments of BWI back to the year of contract closure (interest rate = 2%). As premiums became unfair using this procedure, we continued without assuming an interest charge. See the online supplementary file, Table S8 for the respective results.

^11^ Please see Section 3, ‘Data’ for a detailed explanation of both the yield dataset and the high-resolution rainfall grid used to design the rainfall index. The latter allows an individual matching between farm yield and historical rainfall.

^12^ Our results are robust to changes in the premium rate loading factor. Results assuming a premium rate load of 20% can be found in the online supplementary file, Table S7.

1^3^ Heterogeneous site, e.g. soil, due to conditions across the region, a lack of precipitation was not sufficient to trigger drought losses for 17 farms.

1^4^ We used the rlm function included in the ‘MASS’ package in the statistical software environment R.

1^5^ Note that we test for differences in the prospect value and thus for differences in the insurance demand of decision makers that narrowly frame their insurance as a stand-alone investment. The narrow framing implies that premium subsidies are required to increase the prospect value up to a point where it is positive to make farmers purchase insurance.
