# Supplementary material for: Behavioral weather insurance: Applying cumulative prospect theory to agricultural insurance design under narrow framing
Source: PLoS One. 2020 May 1;15(5):e0232267. doi: 10.1371/journal.pone.0232267 (PMC7194365; doi:10.1371/journal.pone.0232267)
Supplement: S1 Formula — (DOCX) [file pone.0232267.s004.docx]

**S9: Incremental risk premium**

Based on Kim et al. (2014), the framework presented here shows the risk premium of quantile *k*, i.e. the willingness to pay to eliminate a risk in the k-th quantile and move the probability mass of quantile k to the central mean $M_{1}$, according to expected utility preferences $U\left( . \right)$. $F\left( b_{k} \right)$ indicates the upper probability bound. $m_{kn}$ is the n-th moment, i.e. mean, variance or skewness, in the k-th quantile.

$$\Delta R_{k}\approx$$

$$-\left( \frac{1}{2} \right)\frac{U^{''}\left( m_{k1} \right)}{\sum_{i=1}^{K} \{\left[ F\left( b_{i} \right)-F\left( b_{i-1} \right) \right]\cdot U^{'}(m_{i1})\}}\cdot\left[ F\left( b_{k} \right)-F\left( b_{k-1} \right) \right]\cdot m_{k2}$$

$$-\left( \frac{1}{2} \right)\frac{U^{''}\left( M_{1} \right)}{U^{'}\left( M_{1} \right)}\cdot[\int_{x\in S_{k}} \left( m_{k1}-M_{1} \right)^{2}dF(x)]$$

$$-\left( \frac{1}{6} \right)\frac{U^{'''}\left( m_{k1} \right)}{\sum_{i=1}^{K} \{\left[ F\left( b_{i} \right)-F\left( b_{i-1} \right) \right]\cdot U^{'}(m_{i1})\}}\cdot\left[ F\left( b_{k} \right)-F\left( b_{k-1} \right) \right]\cdot m_{k3}$$

$$-\left( \frac{1}{6} \right)\frac{U^{'''}\left( M_{1} \right)}{U^{'}\left( M_{1} \right)}\cdot[\int_{x\in S_{k}} \left( m_{k1}-M_{1} \right)^{3}dF(x)]$$

$$\approx$$

$$-\left( \frac{1}{2} \right)\cdot\frac{\frac{-\alpha}{{m_{k1}}^{\alpha+1}}}{\sum_{i=1}^{K} \{\left[ F\left( b_{i} \right)-F\left( b_{i-1} \right) \right]\cdot\frac{1}{{m_{i1}}^{\alpha}}\}}\cdot\left[ F\left( b_{k} \right)-F\left( b_{k-1} \right) \right]\cdot m_{k2}$$

$$-\left( \frac{1}{2} \right)\cdot(-\frac{\alpha}{M_{1}})\cdot\left( m_{k1}-M_{1} \right)^{2}\cdot\left[ F\left( b_{k} \right)-F\left( b_{k-1} \right) \right]$$

$$-\left( \frac{1}{6} \right)\cdot\frac{\frac{(\alpha^{2}+\alpha)}{{m_{k1}}^{\alpha+2}}}{\sum_{i=1}^{K} \{\left[ F\left( b_{i} \right)-F\left( b_{i-1} \right) \right]\cdot\frac{1}{{m_{i1}}^{\alpha}}\}}\cdot\left[ F\left( b_{k} \right)-F\left( b_{k-1} \right) \right]\cdot m_{k3}$$

$$-\left( \frac{1}{6} \right)\cdot\left( \frac{\left( \alpha^{2}+\alpha\right)}{{M_{1}}^{2}} \right)\cdot\left( m_{k1}-M_{1} \right)^{3}\cdot\left[ F\left( b_{k} \right)-F\left( b_{k-1} \right) \right]$$

$$\approx$$

$$\left( \frac{1}{2} \right)\cdot\left[ F\left( b_{k} \right)-F\left( b_{k-1} \right) \right]\cdot$$

$$\left\{ \frac{{{\alpha(m}_{k1})}^{-\alpha-1}}{\sum_{i=1}^{K} \{\left[ F\left( b_{i} \right)-F\left( b_{i-1} \right) \right]\cdot{m_{i1}}^{-\alpha}\}}\cdot m_{k2}+\left( \frac{\alpha}{M_{1}} \right)\cdot\left( m_{k1}-M_{1} \right)^{2} \right\}$$

$$+\left( \frac{1}{6} \right)\cdot\left[ F\left( b_{k} \right)-F\left( b_{k-1} \right) \right]\cdot$$

$$\left\{ -\frac{{(\alpha^{2}+\alpha)m_{k1}}^{-\alpha-2}}{\sum_{i=1}^{K} \{\left[ F\left( b_{i} \right)-F\left( b_{i-1} \right) \right]\cdot{m_{i1}}^{-\alpha}\}}{\cdot m}_{k3}-(\frac{\left( \alpha^{2}+\alpha\right)}{{M_{1}}^{2}})\cdot\left( m_{k1}-M_{1} \right)^{3} \right\}$$
