# Supplementary material for: Behavioral weather insurance: Applying cumulative prospect theory to agricultural insurance design under narrow framing
Source: PLoS One. 2020 May 1;15(5):e0232267. doi: 10.1371/journal.pone.0232267 (PMC7194365; doi:10.1371/journal.pone.0232267)
Supplement: S3 Table — (DOCX) [file pone.0232267.s007.docx]

Table S3. Wilcoxon signed rank test results for changes in expected utility when Adjustment 1 (insure small losses also) (H1, H2 and H3) is revoked – sensitivity analysis two-year contract

| **Coefficient of relative risk aversion** $\boldsymbol{\varphi}$ | | **p-value^a/b^** | | | | |
| --- | --- | --- | --- | --- | --- | --- |
|  |  | H1 | H2 | | H3 | |
|  |  | H_0_: ${eu}_{traditional \varphi}$≤ ${eu}_{no insurance \varphi}$ | H_0_: ${eu}_{behavioral \varphi}$≤ ${eu}_{no insurance \varphi}$ | | H_0_: ${eu}_{behavioral \varphi}$≤ ${eu}_{traditional \varphi}$ | |
|  |  | H_1_: ${eu}_{traditional \varphi}$> ${eu}_{no insurance \varphi}$ | H_1_: ${eu}_{behavioral \varphi}$> ${eu}_{no insurance \varphi}$ | | H_1_: ${eu}_{behavioral \varphi}$> ${eu}_{traditional \varphi}$ | |
|  | 0 | 0.56 | | 0.37 | | 0.48 |
|  | 0.2 | 0.03 | | 0.02 | | 0.38 |
|  | 0.4 | 1.97 $\cdot$10^-4^ | | 8.92 $\cdot$10^-4^ | | 0.57 |
|  | 0.6 | 7.27 $\cdot$10^-4^ | | 7.27 $\cdot$10^-4^ | | 0.62 |
|  | 0.8 | 7.27 $\cdot$10^-4^ | | 1.09 $\cdot$10^-3^ | | 0.57 |
|  | 1.0 | 7.27 $\cdot$10^-4^ | | 1.97 $\cdot$10^-3^ | | 0.53 |

a Low p-values imply a rejection of the null hypotheses stated in H1-H3

b Bonferroni corrected p-values
