# Supplementary material for: Behavioral weather insurance: Applying cumulative prospect theory to agricultural insurance design under narrow framing
Source: PLoS One. 2020 May 1;15(5):e0232267. doi: 10.1371/journal.pone.0232267 (PMC7194365; doi:10.1371/journal.pone.0232267)
Supplement: S6 Table — (DOCX) [file pone.0232267.s010.docx]

Table S6. Wilcoxon signed rank test results for differences in the prospect value using CPT specifications *Boc.1*, *Boc.2* *Boc.3* and *Bab* (H4 – H8) – sensitivity analysis four-year contract

|  | BWI with all Adjustments fulfilled | BWI excluding Adjustment 1  (small losses not insured) |
| --- | --- | --- |
| **Specifications** | **p-value^a,b^**  H_0_: ${pv}_{behavioral}\leq{pv}_{traditional}$  H_1_: ${pv}_{behavioral}$ > ${pv}_{traditional}$ | |
| *H4: Boc.1* | 0.87 | 1.22 $\cdot$10^-4^ |
| *H5: Boc.2* | 0.99 | 3.05 $\cdot$10^-4^ |
| *H6: Boc.3* | 1 | 1.22 $\cdot$10^-4^ |
| *H7: Bou* | 1 | 0.16 |
| *H8: Bab* | 1 | 1.22 $\cdot$10^-4^ |

a Low p-values imply a rejection of the null hypotheses stated in H4-H8

b Bonferroni corrected p-values
